# The Emerging Role of Microglial Hv1 as a Target for Immunomodulation in Myelin Repair

**DOI:** 10.14336/AD.2023.1107

**Published:** 2024-05-07

**Authors:** Yingxin Tang, Xuan Wu, Jiarui Li, Yuanwei Li, Xiaoxiao Xu, Gaigai Li, Ping Zhang, Chuan Qin, Long-Jun Wu, Zhouping Tang, Dai-Shi Tian

**Affiliations:** ^1^Department of Neurology, Tongji Hospital, Tongji Medical College, Huazhong University of Science and Technology, Wuhan, China.; ^2^Department of Neurology, Mayo Clinic, Rochester, MN 55905, USA

**Keywords:** Hv1 Proton Channel, Remyelination, Demyelinating Diseases, Stroke, Spinal Cord Injuries

## Abstract

In the central nervous system (CNS), the myelin sheath ensures efficient interconnection between neurons and contributes to the regulation of the proper function of neuronal networks. The maintenance of myelin and the well-organized subtle process of myelin plasticity requires cooperation among myelin-forming cells, glial cells, and neural networks. The process of cooperation is fragile, and the balance is highly susceptible to disruption by microenvironment influences. Reactive microglia play a critical and complicated role in the demyelination and remyelination process. Recent studies have shown that the voltage-gated proton channel Hv1 is selectively expressed in microglia in CNS, which regulates intracellular pH and is involved in the production of reactive oxygen species, underlying multifaceted roles in maintaining microglia function. This paper begins by examining the molecular mechanisms of demyelination and emphasizes the crucial role of the microenvironment in demyelination. It focuses specifically on the role of Hv1 in myelin repair and its therapeutic potential in CNS demyelinating diseases.

In the central nervous system (CNS), multi-lamellar extension membranes from the oligodendrocytes wrap around the axon and form the myelin [1-3]. Myelin sheath is a high electrical resistance and low capacitance membrane, and the nodes of Ranvier formed by gaps between adjacent myelin sheaths ensure saltatory impulse propagation of action potentials [1-3]. Traditionally, myelin was regarded as an insulating membrane guaranteeing fast and economic conduction of action potential. However, recent research has indicated that myelin also contributes to modulating the action potential conduction velocity, which is crucial for the precision regulation of electrical signals in neuronal networks [4, 5]. Additionally, myelin and oligodendrocyte-derived metabolic support also contribute to the maintenance of axonal energy homeostasis and survival [6]. Furthermore, myelin is not static but exhibits plasticity. Myelin’s effects on axonal conduction and support can be altered or enhanced by neural network activity, leading to changes in neuronal signaling, a phenomenon known as “myelin plasticity” [5, 7]. Overall, the myelin sheath ensures efficient interconnection between neurons and contributes to the regulation of the proper function of neuronal networks.

The maintenance of myelin and the intricate process of myelin plasticity require cooperation among myelin-forming cells, glial cells, and neural networks [1, 6-10]. This cooperation maintains myelin and regulates myelin thickness and length [8, 9, 11]. Unfortunately, this delicate balance is highly susceptible to disruption by environmental factors, and the repair of the balance is also highly susceptible to environmental factors (see detailed information in Fig. 1). In CNS, compared to gray matter, the white matter containing a large proportion of myelinated nerve fibers has a low blood supply and limited collateral circulation [12]. This anatomical feature renders myelin vulnerable to ischemia. Additionally, oligodendrocytes, particularly oligodendrocyte precursor cells (OPCs), are extremely susceptible to ischemia-induced oxidative stress, excitotoxicity, and inflammation [13-16]. Microglia, the critical resident immune cells in the brain, rapidly activated under pathological conditions, transforming from small body cells into phagocytic cell phenotype [17]. They secrete reactive oxygen species (ROS), pro-inflammatory cytokines such as IL-18, IL-6, and IL-1b, and several kinds of chemokines including CCL2 and CCL5, adhesion molecules, as well as anti-inflammatory cytokines [17-20]. Microglia play a crucial and complicated role in demyelination and myelin restoration [9, 14, 21-23]. On one hand, pro-inflammatory microglia release pro-inflammatory cytokines which aggravate the inflammatory response and thus aggravate the death of neurons and oligodendrocytes [23]. ROS and reactive nitrogen species (RNS) produced by activated microglia lead to intra-axonal mitochondrial disturbances, which result in myelin and axon damage [24, 25]. On the other hand, anti-inflammatory microglia have a neuroprotective effect by phagocytizing dead cell debris and releasing neuroprotective factors to support neuronal survival [23, 26, 27].

**Figure 1.**
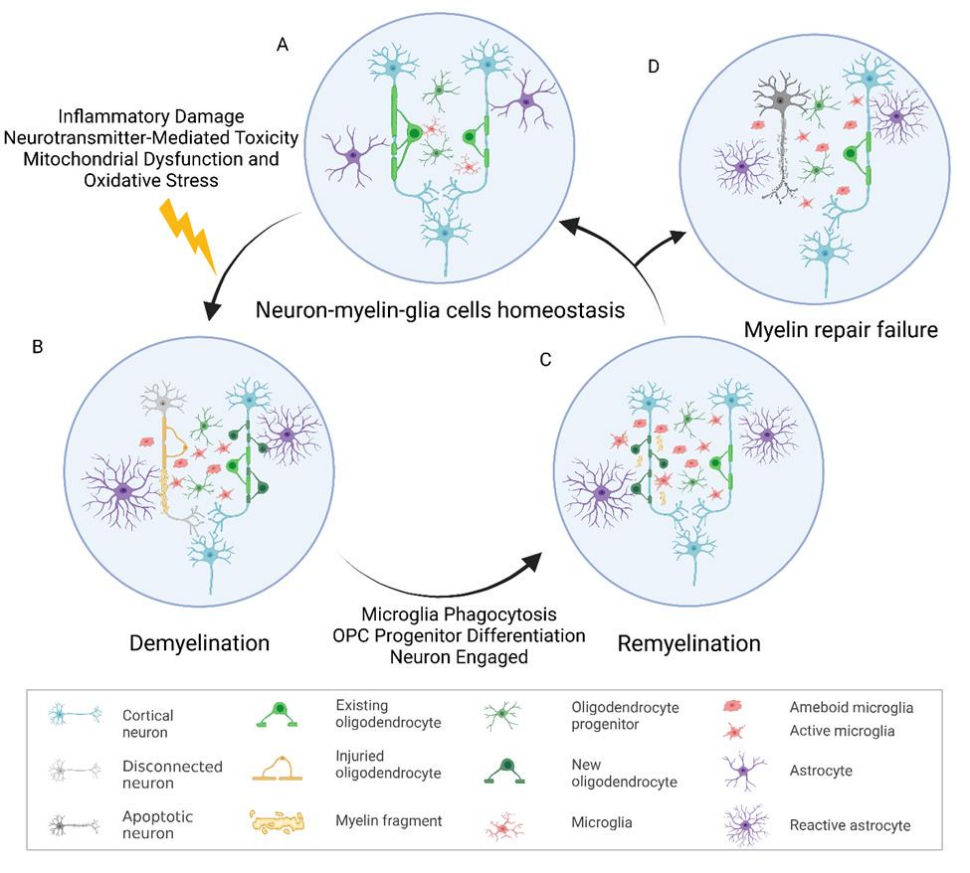
**The maintenance, disruption, reconstruction, and failure repair of myelin**. (**A**) The maintenance of myelin and the intricate process of myelin plasticity require cooperation among myelin-forming cells, glial cells, and neural networks. (**B**) The balance of neuron-myelin-glia cells is highly susceptible to disruption by environmental factors such as inflammatory damage, neurotransmitter-mediated toxicity, mitochondrial dysfunction, and oxidative stress. When homeostasis is disturbed, demyelination occurs. (**C**) CNS initiates spontaneous myelin repair response. This process is mainly involved in microglia phagocytosis, oligodendrocyte progenitor (OPC) differentiation, and neuronal regulation. (**D**) The failure repair of myelin in CNS.

Myelin and its plasticity play a crucial role in various aspects such as learning and memory [28], aging [29], neuronal signaling [30], differentiation of oligodendrocyte precursor cells (OPCs) [31], repair after ischemic stroke [32] and traumatic brain injury [33], secondary lesions after intracerebral hemorrhage [34, 35], as well as many other pathological and physiological conditions. Disruption of myelin architecture is a pathological hallmark of CNS demyelinating disorders, ischemic diseases, traumatic brain and spinal cord injury, neurodegenerative diseases, infectious diseases, seizures, pain, aging, or non-myelinating demyelinated diseases with genetic background [36-42]. Therefore, it is crucial to study how to prevent myelin loss and promote proper myelin repair in various diseases.

As a conserved voltage-gated proton channel, the voltage-gated H^+^ channel Hv1 is enriched in the immune system, rapidly removes protons from depolarized cytoplasm, and is required for producing high-level NADPH oxidase-dependent superoxide in phagocyte. Hv1 is indispensable for maintaining fully functional microglia and plays essential roles in CNS homeostatic and disease pathologies [43-50]. Recent studies have shown that Hv1 plays a role in myelin damage and myelin repair in the CNS [44, 46, 51-53]. However, the complicated functional regulation of Hv1 in microglial during demyelination and remyelination remains largely unknown, which is recognized to be closely related to different cellular phenotypes. This paper focuses on the molecular mechanisms of demyelination, highlighting the crucial role of the microenvironment in demyelination. It also emphasizes the role of Hv1 in myelin repair and its potential therapeutic applications in demyelinating diseases.

## 1. Molecular Mechanisms in Oligodendrocyte Death, Demyelination, and Remyelination

### 1.1 Inflammatory Response

Brain-resident microglia, monocyte-derived macrophages, and astrocytes, important effector cells of inflammatory response in the CNS, play a complex role in multiple demyelinating-related diseases, both in demyelination and remyelination by pro-inflammation and anti-inflammation mechanisms [14, 54-62]. The Janus-faced properties of the inflammatory response and these immune cells, especially microglia, are recognized to be closely related to different cellular phenotypes resulting from different disease-associated microenvironments and unique spatiotemporal patho-physiological patterns of different diseases [17, 60, 63, 64]. For example, both microglia and astrocytes exhibit dual roles towards different types of injuries and different stages of the diseases [65-68]. Pro-inflammatory microglia and A1-reactive astrocytes release pro-inflammatory which aggravate the inflammatory response and thus aggravate the death of neurons and oligodendrocytes [65-68]. In contrast, anti-inflammatory microglia and A2-reactive astrocytes have a neuroprotective effect by phagocytizing dead cell debris and releasing neuroprotective factors to support neuron survival [65-68].

As the crucial resident immune cells of the CNS, microglia are indispensable in both innate and adaptive immunity [69]. In common, microglia serve as immune surveillance cells, monitoring the microenvironment, and clearing pathogens and debris from damaged or dying cells, thus maintaining homeostasis of microenvironments and myelin equilibrium [70-72]. Under pathological conditions, triggered by the disruption of local homeostasis, including the blood-brain barrier, microglia rapidly respond to damage [17, 73]. Excessively activated microglia release pro-inflammatory cytokines, excitatory neurotransmitters, arachidonic acid derivatives, proteinases, and ROS, which induce neuroinflammation [18, 19, 74-77]. TNF-α and C1q, secreted by microglia, directly induce A1 astrocyte dysfunction, resulting in the dysfunction of both oligodendrocytes and neurons [78].

ROS is unstable chemical compounds and mostly exist in a radical form, which means that they contain unpaired electrons on the outer orbital. ROS commonly include superoxide anion (O2-), hydroxyl radicals (OH•), peroxyradicals (ROO•), hydrogen peroxide (H_2_O_2_), and singlet oxygen. Besides, reactive nitrogen species (RNS) like nitric oxide (NO•) can induce oxidative stress too. Electron transport chain leakage in mitochondria and NADPH oxidase (NOX) formation are the two main mechanisms that lead to ROS production within normal functioning cells [79]. Under physiological conditions, moderately induced ROS are pertinent mediators in several normal physiological processes [79-81]. In cellular activities, OH stimulates the activation of guanylate cyclase and the formation of the “second messenger” cGMP in cells and H_2_O_2_ stimulates the activation of transcription factor nuclear factor κB (NF-κB) [79]. In the immune system, ROS serve as anti-microbial agents, which help to combat invading pathogens [80]. ROS are also involved in the formation of long-term potentiation (LTP) and contribute to synaptic plasticity [81]. Normally, ROS is maintained in a dynamic equilibrium. However, CNS has relatively poor protective antioxidant capacity, which means that the CNS is highly vulnerable to excessive ROS [82]. Microglia sense local microenvironments and shift activity states through various sensing receptors, including Toll-like receptors (TLR), complement receptor 3 (CR3; CD11b/CD18), ionic and metabolic purinergic receptors, activated neurotransmitter receptors, and CD36, generating ROS [83]. Excessive ROS produced by microglia are considered to contribute to oxidative stress associated with demyelination, axonal damage, and neuronal loss [19, 24, 25, 74, 84-86].

The relevance of oxidative stress to MS has been confirmed and most studies focused on brain intrinsic cells and recruited monocytes [87]. It is generally accepted that the mechanism of acute demyelinating lesions is the myelin sheaths degraded by phagocytes in the presence of infiltrating T cells [88]. In this model, immune cell recruitment is an early or even initial event [88]. However, other studies hold the contrasting idea that early, focal microglia activation accompanied by extensive oligodendrocyte apoptosis is the major pathological mechanism and peripheral immune cells recruitment is a response to this primary oligodendrocyte pathology [89]. Although tit-for-tat, the above two both hold that T cells, macrophages, and microglia are all activated and recruited in the inflammatory process. A great amount of pro-inflammatory mediators and ROS, such as O2-, OH•, H_2_O_2_, and NO, are generated by infiltrated macrophages, activated microglia, and immature myeloid cells [90].

ROS-mediated tissue injury is critically involved in these inflammatory processes. For example, in a model using cerebellar organotypic cultures stimulated with lipopolysaccharide (LPS), microglia activated by LPS released pro-inflammatory cytokines (IL-1β, IL-6, and TNFα), and increased the expression of inducible nitric oxide synthase (iNOS) and production of ROS [19]. This activation was associated with demyelination and axonal damage in cerebellar cultures. Blocking microglial activation with ethyl pyruvate or allopurinol significantly decreased axonal damage, and to a lesser extent, demyelination. Blocking TNFα significantly decreased demyelination but did not prevent axonal damage and interferon-beta treatment decreased oxidative stress (iNOS and ROS levels) and the release of pro-inflammatory cytokines after LPS stimulation, thus reducing axonal damage [19]. In the chronic-relapsing experimental autoimmune encephalomyelitis (EAE) model of MS, hippocampal microglial cells showed signs of activation, CA1 hippocampal synapses presented an impaired long-term potentiation (LTP) and an alteration of spatial tests became evident. LTP blockade was found to be caused by the ROS-producing enzyme NADPH oxidase [91].

In recent approaches, neurotoxic CNS innate immune populations in EAE mice have been identified by toxic RNA sequencing (Tox-seq). Single-cell RNA sequencing analysis found a specific ROS+ microglia cluster in CD11b+ cells. ROS+ microglia cluster displayed low levels of homeostatic microglia markers (e.g., P2ry12, Sparc, Cx3cr1, and Tmem119) but high levels of oxidative stress and pro-inflammatory genes [e.g., NADPH oxidase subunit 2 (gp91-phox), MhcII, Il1b] [92]. Moreover, in ROS+ microglia and macrophages, several genes of the oxidative stress network were upregulated, such as glutathione transferases (Gsto2 and Gstt2), g-glutathione peroxidase (Gpx7), and the acivicin target genes (Ggt1 and Ggt5) [92]. Treating EAE mice with the compound acivicin, which inhibits the degradation of glutathione, resulted in a decrease in oxidative stress and neurodegeneration in the mice, even when the treatment started 80 days after disease onset [92]. These data suggest that targeting ROS production in innate immune cells is a promising strategy to treat active chronic neuroinflammation, such as that occurring in people with progressive MS [93].

Furthermore, immuno-histochemical data of post-mortem human cerebellar grey matter (GM) from MS and control subjects showed a reduction in myelin and neuronal markers in GM of MS patients, coupled with an increase in the expression of a microglial marker. Superoxide dismutase (SOD1 and 2) enzymes, localized within cerebellar neurons, are up-regulated, yet the activation of subsequent enzymes responsible for the detoxification of hydrogen peroxide, catalase, and glutathione peroxidase are relatively deficient [94].

All these results foster the view that ROS is critically involved in autoimmune-mediated tissue damage in MS. Like MS, neurodegenerative diseases such as Alzheimer’s disease (AD) and Parkinson’s disease (PD) are also characterized by demyelination, axonal damage and neuronal degeneration and death in specific regions of the CNS, in which neuroinflammation is crucial in the onset and the progression of neurodegeneration [95].

ROS has also been implicated in brain injury after ischemic stroke. Mitochondria overproduce ROS immediately after acute ischemic injury, which not only contributes to the injury of macromolecules but also triggers various signaling pathways. ROS activate several pathways, including PI3-K, MAPK, and p53 pathways [96-98], which crosstalk with the death-receptor-mediated extrinsic pathway and then cause further tissue damage. Moreover, the rapid restoration of blood flow increases the level of tissue oxygenation and accounts for a second burst of ROS generation, which leads to reperfusion injury [97].

Moreover, microglia also have important effects on synaptic function. In EAE mice, activated microglia produces large amounts of TNF-α, which induces the increase of glutamate. At the same time, microglial signals are amplified by astroglial ATP, which also promotes glutamate release from astroglia. Excessive glutamate leads to synaptic dysfunction in currents, transmission, and plasticity [99].

Apart from the crucial inflammatory factors, cytokines, and chemokines mentioned previously, iron is another key factor in the demyelination process. The accumulation of iron in gray matter structures has been detected by MRI studies in MS patients [100]. The mechanisms of iron in MS pathogenesis are complex, such as (1) iron could amplify the activated state of microglia resulting in the increased production of pro-inflammatory mediators; (2) excess intracellular iron deposits could promote mitochondria dysfunction; and (3) improperly managed iron could catalyze the production of damaging ROS [101, 102]. However, iron maintains the integrity of oligodendrocytes and myelin, and facilitates their regeneration following injury [103].

Moreover, adaptive immunity also plays a crucial role in demyelination. Microglia are the main antigen-presenting cells (APC) in CNS and play an important role in adaptive immunity by modulating T-cell function [104]. Studies from experimental autoimmune encephalomyelitis (EAE) mice showed that microglia take up myelin antigens and then restimulate encephalitogenic T cells in the CNS through MHC molecules and costimulatory molecules [104]. MHCs, co-stimulatory molecules, and different T-cell polarising factors, the three main signals of lymphocytic activation through the antigen presentation process, increase specifically in EAE [104].

To sum up, microglia mainly exert detrimental functions in demyelinating diseases and damage myelin severely. However, microglial activation also plays a beneficial role in remyelination. A study showed that microglia depletion exacerbates demyelination and impairs remyelination [105]. The microglial beneficial function first comes from its phagocytotic ability. Invoked through receptors including TREM2, complement receptor 3, and signal regulatory protein-α, microglia phagocytose myelin debris in MS plagues [106], which helps to alleviate inflammation and is good for remyelination. Blockage of TREM2 results in increased demyelination in EAE [107]. The microglial beneficial function also comes from the ability to secrete neuroprotective molecules and anti-inflammatory cytokines. Neuroprotective molecules, including insulin-like growth factor, trypsinogen, and fibroblast growth factor, generated by activated microglia promote neurogenesis [108]. Anti-inflammatory molecules from microglia, for example, IL-4 enhances oligodendrogenesis in EAE model [109] and activin A drives oligodendrocyte differentiation [110]. Interestingly, pro-inflammatory cytokine TNF released by microglia also has neuroprotective functions in EAE [111]. In a word, microglia play an important and diverse role in pathological mechanisms of demyelinating diseases, and extensive additional studies are needed to explore its function.

### 1.2 Neurotransmitter-Mediated Toxicity

Different from other organ systems, the efficient interconnection between neurons ensures the powerful function of neuronal networks in the CNS. Chemical synapses and neurotransmitters bridge CNS neurons and mediate communication between different neurons [112-115]. Moreover, several common neurotransmitters also assume the function of communication between neurons and glial cells, which is crucial for the proper function of neuronal networks and synaptic plasticity [116-119]. However, excessive neurotransmitters or anachronistic neurotransmitters mediate cytotoxicity under pathological conditions [120-125].

Glutamate is one of the major excitatory neurotransmitters that mediate cell death in oligodendrocytes and OPCs in diverse CNS injuries [126]. Oligodendrocytes are sensitive to glutamate-induced cell death [127]. Oligodendrocytes mainly express ionotropic glutamate receptors: α-amino-3-hydroxy-5-methyl-4-isoxazolepropionic acid (AMPA), kainite, and N-methyl-d-aspartate (NMDA) receptors [128, 129]. And OPCs strongly express metabotropic glutamate receptors [130]. In the physiological state, glutamate transporters (GluTs) uptake glutamate from the extracellular space and maintain the low extracellular glutamate levels. However, under conditions of energy failure, GluTs on oligodendrocytes, astrocytes, and microglia operate in reverse and release glutamate into the extracellular space, inducing glutamate excitotoxicity [127, 131]. Na^+^/Ca^2+^ channels on oligodendrocytes are opened in the condition of glutamate receptor overstimulation, allowing the influx of Ca^2+^ and cell membrane depolarization [132]. Ca^2+^ also can be released from the endoplasmic reticulum and/or mitochondria upon glutamate stimulation in oligodendrocytes [133]. In addition, the glutamate surge may reduce the capacity of the Na^+^ gradient-dependent antiporter to remove intracellular Ca^2+^ [132]. All of these mechanisms might contribute to the accumulation of cytosolic Ca^2+^, which in turn triggers oligodendrocyte toxicity and results in demyelination and white matter damage [134].

Similar to glutamate, ATP triggers oligodendrocyte excitotoxicity via activation of ionotropic P2X and metabotropic P2Y purinoreceptors on oligodendrocytes [135]. During ischemia, ATP-mediated toxicity to oligodendrocytes occurs mainly via P2X7 receptor subtypes, which induces cell death, myelin damage, and white matter injury [136]. Blockade of P2X7 receptors attenuated post-ischemic injury to white matter [128]. In the situation of ischemia hypoxia, ATP is released extracellularly from glial cells, oligodendrocytes, and even dying cells, leading to the depolarization of mitochondria and the release of ROS [137]. More than 80% of total ATP was produced in mitochondria through the oxidative phosphorylation (OXPHOS) complexes embedded in the inner mitochondrial membrane. In the tricarboxylic acid cycle, NADH and FADH2 are produced and oxidized by complexes I and II [138]. The resulting electrons are passed to molecular oxygen, the final electron acceptor. Furthermore, an electrochemical gradient across the inner membrane was generated by pumping H^+^ ions into the intermembrane space, thereby driving ATP synthesis [139]. ATP together with several enzymes and secondary messengers such as interleukin-1β, activated mitogen-activated protein kinase (MAPK), and nuclear factor-κB, released from oligodendrocytes and other cells mediate oligodendrocyte toxicity and subsequently leads to apoptosis or necrosis of oligodendrocyte [140].

Besides, researchers found that some other neuromediators, such as kinins [141], dopamine [142], adenosine [143], and GABA [144] are implicated in the ischemic damage of oligodendrocytes.

Different from demyelinating disorders, the brain with ischemic injury, such as white matter stroke (WMS), shows a failure to remyelinate, which makes partially damaged white matter tracts progress towards greater damage [145-148]. One potential cause of the remyelination deficits associated with WMS is the specific disruption of the proliferating OPCs to mature into oligodendrocytes by inhibiting myelin-associated proteins and proteins involved in Nogo receptor (NgR1) signaling [149-151].

### 1.3 Mitochondrial Dysfunction and Oxidative Stress

As the power generator of cells, mitochondrial dysfunction plays an important role in the pathogenesis of various demyelinating disorders [152, 153]. Normal OXPHOS reaction produces ROS including superoxide (O2-), hydroxyl radicals (OH), and hydrogen peroxide (H_2_O_2_), which is critical for redox signaling. Under stressful conditions, excess ROS can be generated in oligodendrocytes [19], which is particularly toxic to mitochondria as they interact with and block several key proteins of the respiratory chain [25, 154]. Because of the decreasing capacity of mitochondria, oligodendrocytes and axons become energy deficient, giving rise to intracellular Ca^2+^ influx and Ca^2+^ release from mitochondria [154]. It has also been demonstrated that many pro-inflammatory cytokines are released from injured mitochondria, inducing oligodendrocyte damage [76]. Besides, O2- and NO radicals also lead to the death of oligodendrocytes by lipid peroxidation and the formation of additional ROS [155]. Thus, oxidative stress and mitochondrial dysfunction are both critical players in the pathophysiology of demyelination.

Moreover, excessive ROS and reactive nitrogen species (RNS) produced by activated microglia lead to intra-axonal mitochondrial disturbances, which are elevated and sustained in various neurological disorders, usually resulting in myelin and axon damage [19, 24, 25, 74, 84-86].

## 2. Voltage-Gated Proton Channel Hv1 in CNS

### 2.1 Localization and Functions of Hv1 in CNS

The voltage-gated proton channel Hv1 (the product of the HVCN1 gene; also called voltage-sensor domain-only protein, VSOP) is a dimer of two voltage-sensing domains (VSDs), and unlike other typical voltage-gated ion channels, the Hv1 channel lacks an independent homologous pore domain [156]. Each subunit contains a voltage sensor (S4), and a gate (S1) and forming its own ion channel [157]. The subunits of Hv1 form a dimer interface along the length of S1, and there are also intersubunit contacts between S1 and S4 [158]. These interactions exert a strong effect on gating, particularly on the stability of the open state [158, 159]. Hv1 channels are expressed on the plasma membrane and phagosomes and are widely distributed in many different tissues and cell types [160, 161]. Hv1 channels are mainly expressed in immune cells such as neutrophils, macrophages, B-lymphocytes, and T-lymphocytes, and play an important role in the immune system [161-165]. As a voltage-gated ion channel, Hv1 opens at relatively positive transmembrane voltages and is perfectly selective for protons [166, 167]. The gating voltage of the Hv1 channel is strongly regulated by the transmembrane proton gradient ΔpH, which means that Hv1 opens only when the electrochemical driving force is directed outward, thereby generating an outward H+ current to extrude acid [167, 168]. This characteristic ensures that Hv1 plays an indispensable role in the maintenance of pH homeostasis during high-consumption proton biochemical processes [167]. Hv1 channels rapidly extrude acid and cooperate with the NOX to produce the NOX-dependent superoxide, which is crucial for the phagocyte respiratory burst [169]. Moreover, the Hv1 is involved in the regulation of cell activation and signal transduction in the immune cells, such as the B cell antigen receptor (BCR) signaling in B cells and the TLR9 activation in plasmacytoid dendritic cells [170, 171]. In general, the core functions of Hv1 are acid extrusion after acid loading in cells, cell volume regulation, and resting membrane potential setting [167].

**Table 1:** The major experimental CNS research papers related to the Hv1 channel.

Title	Year	Methods	Results	Comments	Ref.
**Voltage-gated proton channel is expressed on phagosomes**	2009	Hv1 gene-trap and WT mouse Immunoblotting Immunohistochemistry Electrophysiology	Hv1 was expressed in microglia in adult mice The level of microglial Hv1 expression is age-dependent, the neonatal brain did not show the positive signal of Hv1 Hv1 is expressed on phagosomes in neutrophils	The level of microglial Hv1 expression is age-dependent The neonatal brain (9 days) of mice did not show the expression of Hv1	[179]
**The voltage-gated proton channel Hv1 enhances brain damage from ischemic stroke**	2012	*Hv1*^-/-^ and WT mice Middle cerebral artery occlusion (MCAO) Whole-cell patch-clamp recordings in microglia in brain slice Confocal imaging, time-lapse ROS detection, and ATP-induced chemotaxis Magnetic resonance imaging (MRI) Positron emission tomography (PET) imaging *In situ* ROS detection	Hv1 was expressed in microglia The level of microglial Hv1 expression has species differences, Hv1 currents are much larger in mice than in rats Presence of Hv1 protein and Hv1 current in cultured human microglia Activating Hv1 leads to NOX-dependent ROS production in brain microglia. ROS production is significantly attenuated in *Hv1*^-/-^ mice, and *Hv1*^-/-^ mice are protected from NOX-mediated neuronal death and brain damage after stroke	The Hv1 is expressed in human microglia Activating Hv1 leads to NOX-dependent ROS production in brain microglia Knockout Hv1 decreases the ROS and protects brain from excessive oxidative stress	[181]
**Zn^2+^ induces apoptosis in human highly metastatic SHG-44 glioma cells, through inhibiting activity of the voltage-gated proton channel Hv1**	2013	Tumor xenograft SHG-44 glioma cells	The inhibition of Hv1 activity via Zn^2+^ ions can effectively retard the cancer growth and suppress the cancer metastasis	Zn^2+^ is the inhibitor of Hv1 The Zn^2+^ is less specific to Hv1	[259]
**Molecular determinants of Hv1 proton channel inhibition by guanidine derivatives**	2014	Oocytes from *Xenopus laevis* Electrophysiological measurements Mapping tested residues on the Hv1-CiVSP chimera	Identify four Hv1 residues involved in the binding with guanidine derivatives Identify a modified version of 2GBI that can reach the binding site on Hv1 from the extracellular side of the membrane	Several guanidine derivatives are the inhibitors of Hv1 These guanidine derivatives are less specific to Hv1 These guanidine derivatives are unable to permeate cytoplasmic membranes due to polarity, which limits their application	[257]
**Microglial Hv1 proton channel promotes cuprizone-induced demyelination through oxidative damage**	2015	*Hv1*^-/-^ and WT mice Cuprizone demyelination model Immunofluorescence BrdU incorporation and detection of oligodendrocyte progenitor cell (OPC)	*Hv1*^-/-^ mice are partially protected from cuprizone-induced demyelination and motor deficits *Hv1*^-/-^ mice in cuprizone-induced demyelination have reduced ROS production, ameliorated microglial activation, increased OPC proliferation, and increased the number of mature oligodendrocytes	Knockout Hv1 decreases the ROS production in cuprizone-induced demyelination and protects from motor deficits	[180]
**Activation of acid-sensing ion channels by localized proton transient reveals their role in proton signaling**	2015	Optogenetic tool Sniffer patch Live-cell imaging of primary neurons and HEK293T cells	Hv1 can target neuronal ASICs by extruding acids	The existence of proton signaling in the nervous system	[195]
**Clozapine and olanzapine inhibit proton currents in BV2 microglial cells**	2015	Mouse microglial BV2 cell line Electrophysiological recordings	Clozapine and olanzapine inhibit proton currents in BV2 microglial cells	Clozapine and olanzapine exert antioxidant function by inhibiting HV1	[256]
**The Hv1 proton channel responds to mechanical stimuli**	2016	Channel expression in Xenopus laevis oocytes by plasmid transfection Patch clamp recordings Mechanical stimulation of membrane patches	Membrane stretch facilitates Hv1 opening With the presence of a transmembrane pH gradient, membrane stretch alone opens the channel without strong depolarizations	Hv1 responds to mechanical stimuli	[263]
**Deficiency in the voltage-gated proton channel Hv1 increases M2 polarization of microglia and attenuates brain damage from photothrombotic ischemic stroke**	2016	*Hv1*^-/-^ and WT mice Photothrombotic cortical ischemia Primary microglial cultures Immunohistochemistry BrdU incorporation and detection of the proliferation of microglia Western blotting (WB)	*Hv1*^-/-^ mice were partially protected from brain damage and motor deficits after photothrombotic cortical ischemia. Hv1 deficiency was found to shift the M1/M2 polarization in *Hv1*^-/-^ mice and primary cultured microglia	Hv1 has functions to alter microglia polarization phenotype under pathological condition Knockout Hv1 promotes M2 polarization in microglia	[182]
**Unconventional role of voltage-gated proton channels (VSOP/Hv1) in regulation of microglial ROS production**	2017	*Hv1*^-/-^ and WT mice Primary culture of microglia Extracellular ROS measurement Immunocytochemistry and phalloidin staining of microglia Real-time polymerase chain reaction (qPCR) WB tMCAO	ROS production is drastically enhanced in isolated Hv1-deficient microglia in primary culture, probably due to the alteration of actin dynamics and the change of intracellular distribution of cytosolic NADPH oxidase subunit p67 in Hv1-deficient microglia The expression levels of antioxidant genes in the cerebral cortex in *Hv1*^-/-^ mice were age-dependent, the expression levels of antioxidant genes in *Hv1*^-/-^ mice showed slightly decreased at younger and drastically increased at the aged stage The neuroprotective effect of *Hv1*^-/-^ mice on infarct volume depended on the age of animals	ROS production enhanced in primary cultured Hv1-deficiency microglia The neuroprotective effect of *Hv1*^-/-^ mice on infarct volume is age-dependent, the neuroprotection due to HV1 deficiency is more significant in aged mice	[50]
**Hv1 proton channel facilitates production of ROS and pro-inflammatory cytokines in microglia and enhances oligodendrocyte progenitor cells damage from oxygen-glucose deprivation in vitro**	2018	Primary culture of Hv1-deficiency and WT microglia and OPC Oxygen-glucose deprivation (OGD) qPCR Enzyme linked immunosorbent assay (ELISAs) Immunocytochemistry, TUNEL staining, and EdU staining WB	The levels of OGD-induced ROS and pro-inflammatory cytokine production were lower in Hv1-deficient microglia Following OGD, OPCs co-cultured with *Hv1*^-/-^ microglia had attenuated apoptosis and greater proliferation and differentiation with decreases in extracellular signal-regulated kinase 1/2 and p38 mitogen-activated protein kinase phosphorylation	Hv1 facilitates the production of ROS and pro-inflammatory cytokines in primary microglia and enhances OPC damage from OGD *in vitro*	[51]
**Mechanistic insight into the suppression of microglial ROS production by voltage-gated proton channels (VSOP/Hv1)**	2018	*Hv1*^-/-^ and WT mice Microglia and macrophages primary culture Measurement of extracellular ROS production qPCR	ROS production in Hv1-deficient primary cultured microglia is higher than in WT microglia when the cells were exposed to LPS This increase in ROS production in Hv1-deficient cells was not observed in macrophages	HV1 in primary cultured microglia has a different regulatory mechanism for ROS than macrophages and neutrophils	[187]
**Aging alters Hv1-mediated microglial polarization and enhances neuroinflammation after peripheral surgery**	2020	Adult and aged WT mice Tibial fracture Brain slices immunofluorescence WB	Aged mice have upregulated Hv1 and NADPH expression compared with adult mice The percentage of CD16/32-positive M1 microglia colabeling with Hv1 was higher in aged mice after tibial fracture surgery	Hv1/NADPH oxidase upregulation in the aged brain may affect the microglial activation toward M1 polarization and exaggerate postoperative neuroinflammatory responses after peripheral surgery	[196]
**Microglial Hv1 proton channels promote white matter injuries after chronic hypoperfusion in mice**	2020	*Hv1*^-/-^ and WT mice Bilateral common carotid artery stenosis (BCAS) Immunofluorescent staining BrdU WB Transmission electron microscopy Eight-arm radial maze test. Primary co-culture of microglia and OPC OGD qPCR	Hv1 deficiency attenuated BCAS-induced disruption of white matter integrity and impairment of working memory Compared with WT mice, *Hv1*^-/-^ mice exhibited reduced ROS generation, decreased pro-inflammatory cytokines production, and an M2-dominant microglial polarization Hv1-/- mice exhibited enhanced OPC proliferation and differentiation Primary co-culture of microglia and OPC suggested that PI3K/Akt signaling was involved in Hv1-deficiency-induced M2-type microglial polarization and concomitant OPC differentiation	Hv1 is a promising therapeutic target for reducing ischemic WMI and cognitive impairment	[53]
**The voltage-gated proton channel Hv1 contributes to neuronal injury and motor deficits in a mouse model of spinal cord injury**	2020	*Hv1*^-/-^ and WT mice spinal cord injury (SCI) Basso mouse scale (BMS) Live spinal cord slices and whole-cell patch clamp recording Hematoxylin-eosin (H&E) staining Immunofluorescence staining Measurement of ROS and cytokine array	*Hv1*^-/-^ mice showed significant white matter sparing and improved motor recovery The improved motor recovery in *Hv1*^-/-^ mice was associated with decreased interleukin-1β, reactive oxygen/nitrogen species production, and reduced neuronal loss. Deficiency of Hv1 directly influenced microglia activation as noted by a decrease in microglia numbers, soma size, and reduced outward rectifier K^+^ current density	Hv1 may be a promising potential therapeutic target to alleviate secondary damage following SCI caused by microglia/macrophage activation	[251]
**Scorpion toxin inhibits the voltage-gated proton channel using a Zn^2+^-like long-range conformational coupling mechanism**	2020	Venom and toxin RP-HPLC purification Recombinant plasmid construction of recombinant toxins Transfection of HEK293T and ND7/23 cells Primary culture of dorsal root ganglion (DRG) neurons Whole-cell patch clamp recording Homology modeling and docking of toxins and Hv1	The scorpion toxin AGAP (anti-tumor analgesic peptide) potently inhibited HV1 currents	The Zn^2+^ binding pocket in the Hv1 channel might be a hotspot for modulators and valuable for designing HV1 channel ligands	[258]
**Deficiency of microglial Hv1 channel is associated with activation of autophagic pathway and ROS production in LPC-induced demyelination mouse model**	2020	*Hv1*^-/-^ and WT mice Two-point stereotaxic LPC injection to model LPC-induced demyelination Morris water maze Luxol fast blue stain Electron microscopy Immunofluorescence and confocal imaging WB	*Hv1*^-/-^ mice showed reduced myelin damage Hv1 knockout showed reduced ROS and autophagy of microglia in the demyelination region	Microglial Hv1 deficiency ameliorates demyelination through inhibition of ROS-mediated autophagy and microglial phenotypic transformation	[184]
**Proton extrusion during oxidative burst in microglia exacerbates pathological acidosis following traumatic brain injury**	2021	*Hv1*^-/-^ and WT mice Controlled cortical impact injury (CCI) Extracellular pH recordings Measurement of ROS in brain tissue Flow cytometry and ex vivo measurement of membrane potential, intracellular pH level, and ROS production Applying a colony stimulating factor 1 receptor (CSF1R) inhibitor, PLX5622 qPCR WB Beam walk Catwalk XT automated gait analysis Morris water maze Y-maze test Novel object recognition (NOR) Three chamber social interaction Cresyl violet staining	Intracellular pH in microglia and extracellular pH surrounding the lesion site are significantly reduced for weeks after CCI Microglia proliferation and production of ROS were increased during the first week Microglia depletion by PLX5622, markedly decreased extracellular acidosis, ROS production, and inflammation Hv1 promotes oxidative burst activity and acid extrusion in microglia Hv1-deficiency microglia showed reduced ability to generate ROS and extrude protons. *Hv1*^-/-^ mice exhibited reduced pathological acidosis and inflammation after CCI, as well as better long-term neuroprotection and functional recovery	The microglial Hv1is an important link that integrates inflammation and acidosis after CCI	[183]
**Heterogeneity of microglial proton channel in different brain regions and its relationship with aging**	2021	*Hv1*^-/-^ and WT mice qPCR Brain protein carbonyl assay Behavioral test battery Immunohistochemistry and morphological analysis of microglia Transcriptome analyses	Hv1 shows brain region-dependent heterogeneity of gene expression with the highest level in the striatum The age-dependent impact of Hv1 on oxidative stress, microglial morphology, and gene expression profile is more obvious in the cortex than in the striatum *Hv1*^-/-^ mice specifically showed a marked difference in behavior of a battery of behavioral tests in light/dark transition tests only at aged stages	Microglia Hv1 expression is brain region heterogeneous and age-dependent	[218]
**The voltage-gated proton channel Hv1 promotes microglia-astrocyte communication and neuropathic pain after peripheral nerve injury**	2021	Hv1^-/-^/CX3CR1^GFP/+^ mice Lumbar 4 spinal nerve transection (SNT) surgery Mechanical allodynia Thermal hyperalgesia Tail flick test The rotarod test Live spinal cord slices and whole-cell patch clamp recording WB qPCR Drug administration (DHE, IFN-γ, and IFN-γ neutralizing antibody, sulforaphane (Calbiochem), BrdU) In situ ROS detection Fluorescent immunostaining	Hv1 was significantly increased after SNT *Hv1*^-/-^ mice display attenuated pain hypersensitivities, microglial production of ROS, and subsequent astrocyte activation after SNT IFN-γ expression was compromised in spinal astrocytes in *Hv1*^-/-^ mice	Hv1 contributes to microglial ROS production, astrocyte activation, IFN-γ upregulation, and subsequent pain after SNT Hv1 is a novel therapeutic target for alleviating neuropathic pain	[47]
**The voltage-gated proton channel Hv1 plays a detrimental role in contusion spinal cord injury via extracellular acidosis-mediated neuroinflammation**	2021	Adult female and male C57/BL6 mice *Hv1*^-/-^ mice Spinal cord contusion model Basso mouse scale Extracellular pH recordings of mice spine L-Lactate assay Measurement of ROS in spinal cord tissue WB qPCR Primary cell culture and CD11b+ microglia/macrophages isolation Nanostring analysis Flow cytometry of mice spine tissue Luxol Fast Blue (LFB) staining Cresyl violet staining	Tissue pH levels are markedly lower and tissue ROS levels and expression of Hv1 were markedly increased during the first week after SCI *Hv1*^-/-^ mice significantly improved locomotor function, reduced histopathology, and attenuated tissue acidosis, NOX2 expression, and ROS production at 3 d after SCI Nanostring analysis revealed decreased gene expression of neuroinflammatory and cytokine signaling markers in *Hv1*^-/-^ mice Hv1 deficiency reduced microglia proliferation, leukocyte infiltration, and phagocytic oxidative burst detected by flow cytometry	Hv1 represents a potential target that may lead to novel therapeutic strategies for SCI	[248]
**Neutralization of Hv1/HVCN1 with Antibody enhances microglia/macrophages myelin clearance by promoting their migration in the brain**	2021	Human brain tissue Marmoset brain tissue WT C57BL/6 mice and SD rats *SOD1^G93A^* mice B6SJL female mice R6/2 mice *Hv1*^-/-^ mice G3 *Terc^-/-^* mice COS-7, 293T, and Hela cells Primary culture of microglia culture from P0 SD rat Primary culture of bone marrow derived macrophage (BMDM) from 10-12 weeks male C57BL/6 mice Transfection of plasmid DNA and siRNA Myelin isolation from rats and labeling Virus/LPC/Myelin Injection LPS injury Immunocytochemistry *In vitro* assay for myelin phagocytosis Transwell assay WB qPCR RNA sequencing	In physiological conditions, microglia and BMDM express Hv1 with the highest level among glial cells, and upregulation of Hv1 in microglia/macrophages is presented in multiple injuries and diseases of the CNS Myelin debris accumulation occurs in both the focal lesion and the site where neurodegeneration takes place Both genetic deletion of the Hv1 *in vitro* and neutralization of Hv1 with antibody in the brain *in vivo* promotes the migration of microglia/macrophages Neutralization of Hv1 with antibody in the brain *in vivo* promotes myelin debris clearance by microglia/macrophages	A new role of Hv1 in migration of microglia/macrophages	[52]
**Knockout of microglial Hv1 proton channel reduces neurotoxic A1 astrocytes and neuronal damage via the ROS/STAT3 pathway after spinal cord injury**	2023	*Hv1*^-/-^ and WT mice T10 spinal cord contusion model Primary astrocyte culture and intervention Osmotic pump implantation Anterograde corticospinal tract tracing by injection of biotinylated dextran amine into the sensorimotor cortex Basso Mouse Scale Footprint analysis The inclined plane test The rotarod test The open field test Immunostaining Luxol fast blue staining WB Fluorescence-activated cell sorting qPCR Transmission electron microscopy Cell viability and ROS assays Glutamate uptake assay Phagocytosis assay	After SCI, astrocytes proliferated and activated in the peri-injury area and exhibited an A1-dominant phenotype Hv1 knockout reduced neurotoxic A1 astrocytes and shifted the dominant phenotype of reactive astrocytes from A1 to A2, enhancing synaptogenesis promotion, phagocytosis, and neurotrophy of astrocytes with synaptic and axonal remodeling, and motor recovery after SCI Exogenous and endogenous ROS in astrocytes after SCI were reduced by Hv1 knockout *In vitro* results showed that inhibition of ROS reduced the neurotoxic A1 phenotype in primary astrocytes via the STAT3 pathway *In vivo* results showed that the application of the ROS scavenger N-acetylcysteine reduced SCI-induced neurotoxic A1 astrocytes	Microglial Hv1 knockout promotes synaptic and axonal remodeling in SCI mice by decreasing neurotoxic A1 astrocytes and increasing neuroprotective A2 astrocytes via the ROS/STAT3 pathway. Hv1 is a promising target for the treatment of SCI	[194]
**Voltage-gated proton channel Hv1 regulates neuroinflammation and dopaminergic neurodegeneration in Parkinson’s disease models**	2023	HV1 expression analysis of PD patients and controls in GEO datasets *Hv1*^-/-^ and WT mice Parkinson’s disease model construction by injection of MPTP and LPS Primary microglial culture and treatment ROS and NO quantification N27 cell culture and conditioned media experiments Immunohistochemistry and immunocytochemistry qPCR	The mRNA expression of *HVCN1* in the brains increased in PD patients, especially in male PD patients Hv1 knockout showed the neuroprotective effects and decreased pro-inflammatory cytokine levels and pro-oxidant factors both in MPTP and LPS Parkinson’s disease models In primary microglial cultures, with LPS treatment increasing Hvcn1 mRNA levels and *Hv1*^-/-^ microglia failing to exhibit the LPS-mediated inflammatory response Conditioned media from *Hv1*^-/-^ microglia treated with LPS resulted in an attenuated loss of cultured dopamine neuron cell viability	Hv1 is upregulated and mediates microglial pro-inflammatory cytokine production in PD models and represents a novel target for neuroprotection in PD	[49]
**Traumatic brain injury-induced inflammatory changes in the olfactory bulb disrupt neuronal networks leading to olfactory dysfunction**	2023	*Hv1*^-/-^ and WT mice CCI Intranasal administration of NOX2 inhibitor qPCR Immunohistochemistry Flow cytometry and *ex vivo* functional assays *In vivo* electrophysiological recording of spontaneous neuronal firing and network activities Buried food test Two-bottle discrimination test Odor memory test Y-maze test	CCI caused a rapid inflammatory response in the olfactory bulbin (OB) as early as 24 h post-injury and sustained for up to 90 days after CCI, including elevated mRNA levels of proinflammatory cytokines, increased numbers of microglia and infiltrating myeloid cells, and increased IL1β and IL6 production in these cells Significant upregulation of the Hv1 and NOX2 expression levels after CCI *In vivo* OB neuronal firing activities showed early neuronal hyperexcitation and later hyponeuronal activity in both the glomerular layer and mitral cell layer after traumatic brain injury, which were improved in the *Hv1*^-/-^ mice In a battery of olfactory behavioral tests, WT/TBI mice displayed significant OD compared with Hv1 KO/TBI or NOX2 KO/TBI mice Intranasal delivery of a NOX2 inhibitor (NOX2ds-tat) ameliorated post-traumatic OD	The importance of OB neuronal networks and its role in TBI-mediated OD Targeting Hv1/NOX2 may be a potential intervention for improving post-traumatic anosmia	[264]

In the CNS, under the steady state, the HV1 channels selectively expressed in the CNS immune cells, mainly dendritic cells and tissue-resident macrophages (including parenchymal microglia, meningeal and perivascular macrophages) [172-177]. These tissue-resident macrophages and dendritic cells play a crucial role in maintaining the homeostasis of the microenvironment under physiological conditions and a variety of immune-related disease processes in CNS [175]. Unlike peripheral immune tissues, the CNS is a compartmentalized organ, with a variety of membranous spacer tissues, including the dura, meninges, choroid plexus, the blood-brain barrier (BBB), and blood-leptomeningeal barrier (blood-CSF barrier), dividing the CNS into different compartments, where pathophysiological responses may be quite different [175, 178]. This physiological property is the structural basis for the maintaining of microenvironmental homeostasis in the CNS, as well as imposing substantial difficulties for CNS research. In 2009, *Hv1*^-/-^ mice were generated by both the Clapham and Okumura laboratories to study the pathophysiological function of Hv1 *in vivo* [169, 179]. Numerous studies using Hv1 knockout mice have demonstrated that microglial Hv1 is critical for NOX-dependent ROS production in demyelinating diseases [180], cerebral ischemia [181, 182], chronic hypoperfusion [53], traumatic brain injury [183], spinal cord injury (SCI) [184], neuropathic pain [185], etc. However, during neuropathology, peripheral blood-derived immune cells infiltrate the CNS, which share most of the immune markers with CNS-resident immune cells and it is difficult to distinguish them from CNS-derived immune cells [172]. Therefore, selective *Hv1* knockout may be a better method to study the role of the Hv1 channel in the CNS. We summarize major experimental CNS research papers related to the Hv1 channel in recent years in Table 1.

### 2.2 Voltage-Gated Proton Channel Hv1 in Microglia

Microglia are innate immune cells of the brain and make up 5%-15% of brain cells [186]. As part of the CNS monocyte-macrophage system, microglia are the only immune cells that are resident in the brain parenchyma, which are in direct contact with neuronal networks and glial cells in the CNS [172, 175]. Microglia actively monitor the microenvironment and are rapidly involved in a wide range of disease pathogenesis [60, 71, 72, 172, 175]. Microglia bear several ion channels, including K^+^ channels, Na^+^ channels, TRP channels, Cl^-^ channels, and proton channels, which play important roles in both physiological and pathological brain functions including proliferation, cytokines and cytotoxic substances production, morphological changes, and migration of microglial cells [45].

**Figure 2.**
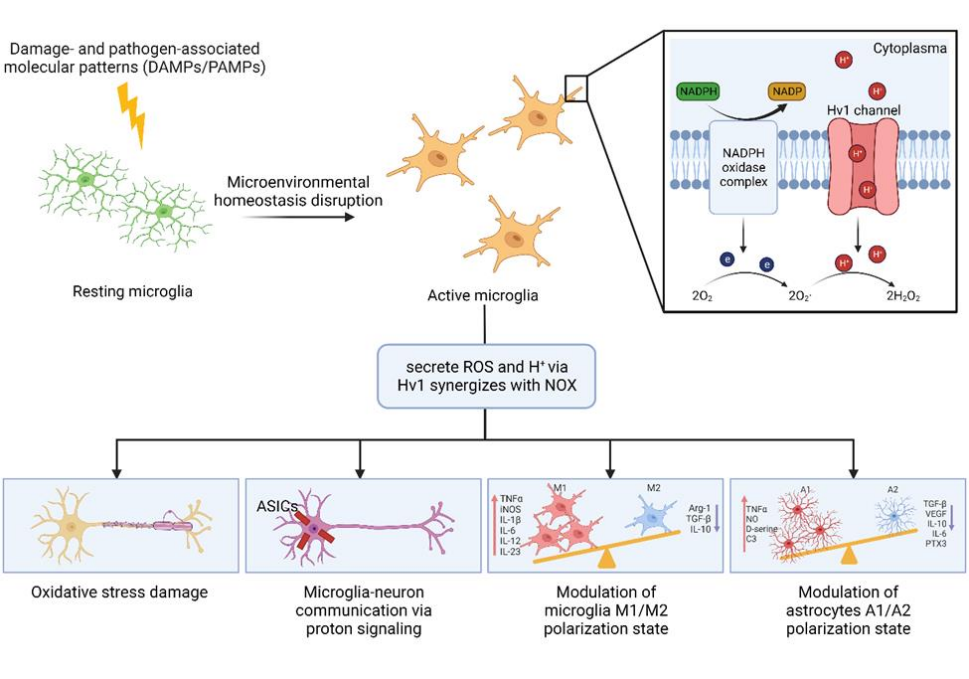
**Microglial Hv1 channel and its multifaceted roles**. Hv1 is required for NADPH oxidase (NOX)-dependent ROS production. Microglia secrete ROS and H^+^ via Hv1 synergizes with NOX. The oxidative stress can lead to demyelination and axonal degeneration. Microglia Hv1 channel mediates the microglia-neuron communication via the extrusion of acids and excitation of neuronal acid-sensing ion channels (ACICs). Microglia Hv1 also contributes to the modulation of microglia and astrocytes polarization.

Similar to its function in peripheral immune tissues, Hv1 has the same multifaceted functions in microglia, with a prominent role in regulating ROS generation [187]. In the CNS, Hv1, and NOX are highly expressed in microglia. As mononuclear phagocytic cells, microglia express high levels of NOX2 (also known as gp91phox), the sole function of which is to generate ROS [188]. Mitochondrial ROS is another source of oxidative stress in microglia, which are the by-products of oxidative phosphorylation. NOX2 consists of a short cytosolic N-terminal domain followed by six transmembrane domains and a long C-terminus that has FAD and NADPH binding sites. NOX2 complex consists of the membrane-bound flavorcytochrome b558 containing subunits p22phox and NOX2 (gp91phox) and the cytosolic regulatory proteins p40phox, p47phox, and p67phox and the small GTPase Rac1 [18, 189]. When NOX is activated by phorbol myristate acetate (PMA), intracellular acidosis is detected in *Hv1*^-/-^ microglia, indicating the cooperative activation of NOX and Hv1 [181]. In addition, compared with that in wild-type (WT) microglia, less ROS is produced and accumulated in *Hv1*^-/-^ microglia in brain slices, which suggests that Hv1 is required for NOX-dependent ROS production of microglia (see detailed information in Fig. 2) [181]. Microglia have been implicated in various pathological conditions, such as MS, AD, PD, ischemic stroke, epilepsy, bacterial meningitis, amyotrophic lateral sclerosis, HIV dementia, neuropathic pain, Huntington’s disease, and Nasu-Hakola disease [190]. Due to the presence of Hv1, one of the major ion channels in microglia, it is theoretically involved in these pathological conditions. Numerous studies using Hv1 knockout mice have demonstrated that microglial Hv1 is critical for NOX-dependent ROS production in demyelinating diseases [180], cerebral ischemia [181, 182], chronic hypoperfusion [53], traumatic brain injury [183], spinal cord injury (SCI) [184], neuropathic pain [185], etc.

Moreover, the Hv1 channel also contributes to the cellular activation and phenotypic transformation of microglia by modulating the production of NOX-dependent ROS, the regulation of the luminal pH, and metabolic reprogramming [182, 191-193]. Studies showed that the M2 polarization of microglia is enhanced after knockout Hv1 [53, 182]. Consequently, the Hv1 channel also regulates microglia communication with other types of cells in the CNS, including neuronal networks and glial cells [193-195]. Zeng WZ et al. showed that Hv1 channels regulated the release of protons, which activated neighboring Neuronal acid-sensing ion channels [195]. Peng J et al. found that *Hv1*^-/-^ mice display attenuated pain hypersensitivities, reactive oxygen species (ROS), and subsequent astrocyte activation after SNT compared with wild-type mice [47]. They induced that microglial Hv1 channel promoted microglia-astrocyte communication and neuropathic pain after peripheral nerve injury [47]. Li Y et al. further stated that knockout Hv1 reduced neurotoxic A1 astrocytes and neuronal damage after spinal cord injury [194].

Meanwhile, the function of the microglial Hv1 channel is closely related to the microenvironment and animal age. Yoshifumi Okochi et al. found that there were no signals of Hv1 expression in the WT neonatal brain (9 days) slices[179]. Kawai et al. found that in contrast to the decrease in ROS production in the CNS of *Hv1*^-/-^ mice *in vivo* and *in vitro* [47, 51, 53, 180-184, 194], ROS production is drastically enhanced in primary cultured *Hv1*^-/-^ microglia [50, 187]. Moreover, the expression levels of antioxidant genes in the cerebral cortex in *Hv1*^-/-^ mice were age-dependent compared to WT mice [50]. The expression levels of antioxidant genes in *Hv1*^-/-^ mice showed slightly decreased at younger and drastically increased at the aged stage [50]. Zhang ZJ et al. also demonstrated that CNS in aged mice have upregulated Hv1 and NADPH oxidase subunit expression compared with adult mice [196]. The upregulation of Hv1 in aged mice may shift the dynamic equilibrium of microglial activation toward M1 polarization and exaggerate neuroinflammatory responses after peripheral surgical intervention [196]. However, in the primary culture of microglia, it should be identified whether the newborn WT mice (P0-2) express Hv1 in the microglia by qPCR or WB.

In summary, Hv1 is highly expressed in microglia in the CNS, serving as one of the major ion channels in resting microglia. Generally, Hv1 is involved in pH regulation and NOX-dependent ROS production, which play significant roles in physiological and pathological processes. Moreover, the Hv1 channels also contribute to the phenotypic transformation of microglia and the intercellular communication between microglia and other cells. Therefore, the complex role of Hv1 in microglia is a potential therapeutic target for the treatment of neurological disorders related to ROS and neuroinflammation. However, Hv1 channels were not specifically knocked down in microglia in most of the studies but rather defaulted that the HV1 channels are only expressed in microglia in the CNS, even under pathological conditions. With the disruption of BBB, blood-derived immune cells, including neutrophils, macrophages, and lymphocytes, where Hv1 channels also highly express, infiltrate CNS, and participate in the inflammatory response with CNS resident immune cells [73, 197, 198]. These blood-derived immune cells behave quite differently from the microglia, and distinguishing the difference may help to understand the pathophysiologic processes [187, 199]. In conclusion, the complex pathophysiological processes in the CNS associated with the Hv1 channels in microglia have not been adequately studied.

### 2.3 Voltage-Gated Proton Channel Hv1 in Neutrophils and Lymphocytes

Neutrophils and lymphocytes, important immune cells, also contribute to CNS demyelination and myelin repair, especially inflammatory demyelinating diseases. Neuropathologic tissue examination identified different types of immune cells in different inflammatory demyelinating diseases. CD4+ and CD8+ T cells are the main cells in MS plaques and neutrophils and eosinophils are mainly in NMO [197, 198].

Pathogenesis of demyelinating diseases is characterized by disruption of the BBB and subsequent infiltration of lymphocytes and activation of microglia, resulting in demyelination and axon degeneration [73]. When activated, myelin-specific lymphocytes outside CNS cross the BBB via the interaction of lymphocyte integrins with cell adhesion molecules on endothelial cells [200]. In addition to the most important binding of the very late antigen-4 integrin on lymphocytes to vascular cell adhesion molecule-1 on brain vascular endothelium [200], different adhesion molecule interactions or chemokines in different inflammatory cell types have been detected, such as α4integrin, ALCAM, ICAM, CCL5, CXCL10, and CCL2/CCR2 by Th1+, Th17+ and CD8+ T cells [201-203] or CXCL12/CXCR4, CCR7/CCL19/TLR4 by monocytes [204, 205]. Neuropathologic tissue examination identified different types of lymphocytes in different demyelinating diseases. CD4+ and CD8+ T cells are the main cells in MS plaques and neutrophils and eosinophils are mainly in NMO [197, 198].

Most studies on animal models of MS validated that MS is caused by recruitment in the CNS of self-reactive lymphocytes, mainly CD4+ T cells [206]. In MS lesions and cerebrospinal fluid of MS patients, high levels of CD4+ T cells (T helper, Th) cells and related chemokines and pro-inflammatory cytokines have been detected. It has been found that Th1, Th17, Th1-like Th17, Th9, and Th22 are associated with MS, which mediate the breakdown of BBB, the activation of resident microglia and astrocytes, and finally the outcome of neuroinflammation [207].

Although most studies on MS have centered on the role of effector CD4+ T cells, accumulating data suggests that CD8+ T cells also play a significant role. It was identified in some cases that plenty of CD8+ T cells contacted with oligodendrocytes or axons closely and polarized granzyme B towards the contact site were detected in CD8+ T cells [208]. In addition, in brain inflammation models induced by oligodendrocyte reactive CD8+ T cells, primary demyelination was detected [209]. These data suggest that cytotoxic T cells (CD8+) may selectively mediate the inflammatory demyelination in MS and other demyelinating diseases [209]. However, HLA-E was detected on astrocytes and endothelial cells in active MS lesions inducing an immunoregulatory phenotype in CD8+ cells, which suggested that at least a subpopulation of CD8+ T cells may play a neuroprotective role [210].

B cells are mainly found in the perivascular and meningeal inflammatory infiltrates and the meningeal inflammation correlates with the extent of active demyelination and neurodegeneration in the underlying cortex [211]. Compared with T cells, B cells are sparse in the MS plague and their function in the MS formation is not clear. But some patients show good responses to therapies, which selectively eliminate circulating B cells [212].

Although Hv1 is also highly expressed in neutrophils and lymphocytes, there is limited research related to the role of Hv1 in neutrophils and lymphocytes in demyelination and myelin repair.

## 3. Hv1 in Central Nervous Demyelination Diseases

### 3.1 Hv1, Demyelination in CNS Aging and Neurodegenerative Diseases

The lesions of myelin are common in the CNS normal aging and neurodegenerative disease [52, 213-215]. The normal nerve fibers are affected so that the normal timing sequences within neuronal circuits break down [215, 216]. Microglia are required for the preservation of myelin integrity by preventing this degeneration in aging and neurodegenerative diseases [9, 217]. Loss of myelin health due to the absence of microglia is associated with the appearance of a myelinating oligodendrocyte state with altered lipid metabolism and this mechanism is regulated through disruption of the TGFβ1-TGFβR1 axis [9]. In these processes, microglia differentiate into different disease-associated phenotypes in the different disease microenvironments and are intimately involved in the diseases [175]. Microglia are involved in the clearance of degenerated myelin debris for accelerating remyelination, but excessively activated microglia engulf healthy myelin sheath for inhibiting remyelination [57]. In amyotrophic lateral sclerosis (ALS), Huntington’s disease, and Parkinson’s disease, there was a trend of increase in Hv1 protein level and upregulated neuroinflammation in the CNS [49, 52]. Moreover, aging alters Hv1-mediated microglial polarization and enhances neuroinflammation after peripheral surgery [196, 218].

### 3.2 Hv1, Demyelination and Myelin Repair in CNS Inflammatory Demyelinating Diseases

Demyelination refers to the loss of myelin around the axons, which is accompanied by neurodegeneration and inflammation. The inflammatory response is key to this demyelinating disease, affecting both the degree of myelin damage and remyelination [219]. Activation of microglia is involved in the pathogenesis of myelin loss. Pathogenesis of demyelinating diseases is characterized by disruption of the BBB and subsequent infiltration of lymphocytes and activation of microglia, resulting in demyelination and axon degeneration [73]. The involvement of microglia in the inflammatory processes of multiple sclerosis (MS) has been demonstrated by TSPO tracers and these microglia are iron-enriched and express a variety of pro-inflammatory cytokines [220, 221]. In the white matter of MS patients, the number of P2Y12^+^ homeostatic microglia significantly reduced, while TMEM119^+^ microglia are predominantly found at the edge of active lesions. These finding further confirms the correlation between microglial activation and MS development [219].

In the pathology of inflammatory demyelinating diseases such as MS, classically activated microglia (CAM) secret ROS and pro-inflammatory cytokines, which promote inflammation and demyelination. However, alternatively activated microglia (AAM) are primarily concerned with anti-inflammatory functions and contribute to oligodendrocytes differentiation and initial remyelination [110, 222]. As mentioned previously, Hv1 is necessary for microglia to produce NOX-dependent ROS and reduce NOX-dependent acidification [180]. ROS is considered to be a mediator of demyelination and axon damage in MS [85]. In addition, autophagy is a conserved intracellular mechanism, in which damaged or dysfunctional proteins, lipids, and organelles are degraded by lysosomes [223]. Autophagy within microglia scavenging of depolarized mitochondria reduced the overproduction of ROS, which had a protective effect on MS [224].

In mouse model of cuprizone-induced demyelination, a model for MS, *Hv1*^-/-^ mice are partially protected from demyelination and motor deficits compared with those in WT mice, which is accompanied by reduced ROS production, ameliorated microglial activation, increased oligodendrocyte progenitor cell (NG2) proliferation, and increased number of mature oligodendrocytes. These results suggest the microglial Hv1 proton channel is a unique target for controlling NOX-dependent ROS production in the pathogenesis of MS [180].

In our recent study, we established a mouse model of cuprizone-induced demyelination (fed 0.2% (w/w) cuprizone combined with powdered chow for 4 weeks) [225], a model for MS, in WT C57BL/6 or *Hv1*^-/-^ mice. Compared with WT mice, *Hv1*^-/-^ mice were partially protected from demyelination and motor deficits, which were accompanied by reduced ROS production, ameliorated microglial activation, increased oligodendrocyte progenitor cell (NG2) proliferation, and increased number of mature oligodendrocytes [180]. We established another model of demyelination by injecting LPC into the corpus callosum at two points in C57BL/6 or *Hv1*^-/-^ mice. Similar to the cuprizone-induced demyelination model, the LPC-mediated myelin damage was reduced by Hv1 deficiency too. Furthermore, we found that the production of ROS and autophagy induced by microglia increased in the demyelination region, which were also inhibited by *Hv1* knockout [84]. These results suggest that the Hv1 proton channel is a unique target for controlling NOX-dependent ROS production in the pathogenesis of MS and microglial Hv1 deficiency ameliorates demyelination through inhibition of ROS-mediated autophagy and microglial phenotypic transformation [84, 180].

### 3.3 Hv1, Demyelination and Myelin Repair in Stroke

Apart from demyelinating disorders, demyelination has been also involved in ischemic diseases, but the mechanisms are not the same.

In ischemic conditions, energy crisis, cytotoxic edema, endothelial dysfunction, ROS generation, and cell depolarization from the breakdown of transmembrane gradients produce a complex cascade resulting in cell damage and death (neurons, glia, oligodendrocytes) [226]. Compared with glial cells and some neurons, oligodendrocytes are more vulnerable to ischemia [227]. Energy crisis and metabolic stress in cerebral ischemia led to excessive excitatory neurotransmitters like glutamate and ATP, which bind to their receptors on the oligodendrocytes plasmalemma and result in a cytosolic Ca^2+^ surge and overload. This oligodendrocyte excitotoxicity ultimately mediates ischemic damage to white matter [13-16]. Oligodendrocytes contain the highest levels of immobilized, protein-bound iron. Energy crisis in cerebral ischemia leads to lactic acidosis, which induces the mobilization of protein-bound iron stores and increases the levels of cytosolic Fe^2+^, resulting in oxidative stress [228]. For supporting myelin membrane and huge volume, oligodendrocytes have the highest rate of oxidative metabolism, which also makes them more vulnerable to ischemia and generates more ROS [229]. So, oligodendrocytes are highly sensitive to various stimuli in cerebral ischemia, including inflammation, oxidative stress, and excitotoxicity [145, 230, 231], all of which contribute to demyelination and deficiencies of remyelination.

NOX-dependent ROS generation in brain microglia is induced by cerebral ischemia and inflicts damage on native cells such as neurons and glia. Reducing NOX-related oxidative stress could ameliorate neuronal damage in ischemic stroke [232]. To detect the mechanism, a recent study investigated the Hv1’s potential role in neuronal damage in ischemic brain injury using the MCAO stroke model [181]. It has been found that the infarct volume (observed by TTC staining in brain slices as well as *in vivo* MRI and PET imaging) in *Hv1*^-/-^ mice was significantly smaller than that in WT mice after MCAO and the *Hv1*^-/-^ mice had significantly better neurological scores than WT mice. Caspase-3 and TUNEL positive neurons significantly decreased in the peri-infarct area of *Hv1*^-/-^ mice than that of WT mice. And pretreatment of NOX inhibitors significantly reduced microglia-induced neuronal death, which suggested that microglial Hv1-dependent neuronal death was likely due to the NOX activation. In addition, TNFα, IL-1β, IL6, IFN-γ, VEGF, glutamate, NO, and ROS, released by microglia during ischemic stroke, only ROS production was also decreased in *Hv1*^-/-^ mice. And ROS scavengers EUK-207 reduced its effects. These data suggest that Hv1 protects brain tissue by reducing NOX-driven ROS during cerebral ischemia [181].

Furthermore, studies have confirmed that activated microglia play a biphasic role in cerebral ischemia, depending on their polarization states [64]. Microglia in classical pro-inflammatory M1-like state play a deleterious role in ischemic stroke, however, microglia in alternative anti-inflammatory M2-like state are critical for attenuating neuronal apoptosis, enhancing neurogenesis, and promoting functional recovery after cerebral ischemia [64]. NOX2 inhibition or suppression of ROS production was shown to shift the microglial polarization from M1 toward M2 state and attenuated brain injury after stroke [233, 234]. We investigated the role of microglial Hv1 proton channel in modulating microglial M1/M2 polarization during the pathogenesis of ischemic cerebral injury using a mouse model of photothrombosis in C57BL/6 or *Hv1*^-/-^ mice. Compared to WT mice, *Hv1*^-/-^ mice were partially protected from brain damage and motor deficits, accompanied by reduced ROS production, and shifted the microglial polarization from M1 to M2 state. Hv1 deficiency was also found to shift the M1/M2 polarization in primary cultured microglia [182]. Taken together, studies suggest that the microglial Hv1 proton channel protects brain tissue by reducing NOX-dependent ROS and modulating microglial M1/M2 polarization in the pathogenesis of ischemic stroke.

During ischemic stroke, microglia release ROS, cytokines, glutamate, and nitric oxide (NO) that increase cell death. Of TNFα, IL-1β, IL6, IFN-γ, VEGF, glutamate, NO, and ROS, only ROS production was reduced in *Hv1*^-/-^ compared to WT both in cultured microglia and brain slices after OGD (oxygen-glucose deprivation). Microglial ROS production was also much lower in *Hv1*^-/-^ mice than in WT mice in MCAO model. In addition, phosphorylation of NF-κB subunit, p65 (P-p65), a critical pro-inflammatory transcription factor affected by ROS signaling in stroke increased significantly in WT mice but not in Hv1^-/-^ mice. Together, these results indicate that Hv1 contributes to microglial activation, ROS production, and NF-κB phosphorylation *in vivo* after stroke [181].

In a mouse model of subcortical vascular dementia via bilateral common carotid artery stenosis, *Hv1*^-/-^ mice were showed to attenuate bilateral common carotid artery stenosis-induced disruption of white matter integrity and impairment of working memory. *Hv1*^-/-^ mice exhibited reduced ROS generation, decreased pro-inflammatory cytokines production, and an M2-dominant rather than M1-dominant microglial polarization. *Hv1*^-/-^ mice also exhibited enhanced OPC proliferation and differentiation into oligodendrocytes, in which PI3K/Akt signaling was involved [53].

Not only in cerebral ischemia but also in ischemic periventricular leukomalacia (PVL) and white matter injury (WMI), microglial Hv1 plays an important role [51]. In a recent OGD study *in vitro*, the levels of OGD-induced ROS and pro-inflammatory cytokine production were dramatically lower in *Hv1*^-/-^ microglia than that in WT microglia. Following OGD, OPCs co-cultured with *Hv1*^-/-^ microglia had attenuated apoptosis and greater proliferation and differentiation than those co-cultured with WT microglia. The decreases in extracellular signal-regulated kinase 1/2 and p38 mitogen-activated protein kinase phosphorylation were involved in the mechanism [51]. These results indicate that Hv1 facilitates the production of ROS and pro-inflammatory cytokines in microglia and enhances damage to OPCs from OGD.

Further studies on the subcortical vascular dementia model using *Hv1*^-/-^ mice have found that Hv1 deficiency attenuated the disruption of white matter integrity and impairment of working memory. Compared with that in WT mice, *Hv1*^-/-^ mice exhibited reduced ROS generation, decreased pro-inflammatory cytokines production and an M2-dominant microglial polarization, and enhanced OPCs proliferation and differentiation into oligodendrocytes. PI3K/Akt signaling was involved in Hv1-deficiency-induced M2-type microglial polarization and concomitant OPCs differentiation. In conclusion, microglial Hv1 and Hv1 mediated ROS generation, proinflammatory cytokines production, and microglial polarization play a key role in myelin injury and thus may be a promising therapeutic target for reducing ischemic WMI and cognitive impairment.

Compared to ischemic stroke, white matter injury, and demyelination after intracerebral hemorrhage (ICH) have been relatively poorly studied. However, post-ICH, white matter injury, and secondary ischemic lesions are also not uncommon [34, 235-237]. White matter lesions after brain hemorrhage are often associated with poor outcomes [238-241]. Neuroinflammation, oxidative stress, and excitotoxicity play a critical role in demyelination, axonal damage, and loss of oligodendrocytes after ICH [34, 242, 243]. In 2008, researchers first found that demyelination occurred inside and at the edge of the hematoma, and subsequently, neurons died [244]. Further study showed that ferroptosis in oligodendrocyte progenitor cells mediates white matter injury after ICH [245]. Interestingly, the researchers found that axonal injury occurred prior to the infiltration of activated microglia into the white matter [244], suggesting that microglia may mediate secondary myelin injury and myelin repair. Moreover, the predominant subpopulation of activated microglia after ICH changes with the course of the disease. The M1 polarization peaks as early as 4 hours after ICH, whereas M2 polarization peaks 24 hours after ICH [246, 247]. This finding suggests that it may be necessary to differentiate the time window of post-ICH demyelination to optimize treatment for its different pathological mechanisms. Zheng et al. used *in vivo* and *in vitro* ICH models and found that in the demyelination process, ICH induced M1 microglia and A1 astrocytes through ROS-induced NF-κB p65 translocation that hindered OPC maturation. However, there are no relevant studies on the role of microglia Hv1 channels in myelin repair after ICH.

### 3.4 Hv1, Demyelination and Myelin Repair in Spinal Cord Injury

It has been noted that the expression of Hv1 increased in animal models of spinal cord injury (SCI). In a mouse model of contusion, Hvcn1 mRNA and Hv1 protein in injured spinal cord tissue were elevated at 3 days after SCI [248]. It was further confirmed by performing bioinformatics analysis that most of the increased Hvcn1 mRNA came from both activated resident microglia and infiltrated macrophages, which contributed to the secondary damage after SCI [249]. To address the function of Hv1 in SCI, recent studies based on a contusion mouse model have found a significant improvement in motor recovery, attenuated neuronal loss, increased white matter sparing, and reduced demyelination in the *Hv1*^-/-^ SCI mice compared to the WT/SCI mice [184, 248, 250, 251].

SCI also induced up-regulation of microglia NOX2 expression [252]. The increased NOX2 activity leads to excessive ROS production, which can disrupt cell signaling, activate the mitochondrial pro-apoptotic signal, and cause oxidative damage to lipids, proteins, and DNA [253]. Inhibition of both ROS and NOX2 showed approved outcomes of SCI [254]. Moreover, NOX-dependent ROS production is significantly attenuated in the *Hv1*^-/-^ SCI mice model compared to the sham model [184, 248, 251]. Collectively, these findings suggest that targeting Hv1 could be a promising approach to control NOX-dependent ROS production and improve outcomes in SCI.

Microglia/macrophage activation and polarization are pathological phenomena after SCI. *Hv1*^-/-^ SCI mice showed fewer activated microglia and smaller cell soma compared to WT SCI mice, indicating that depletion of Hv1 alleviated microglia/macrophage activation after SCI [251]. In addition, in the *Hv1*^-/-^ SCI mice, the proportion of anti-inflammatory makers (CD206, Arginase 1, chitinase-like 3) increased, while the proportion of pro-inflammatory makers (IL-1β, TNF-α, Cxcl10, CD68, Nos2, ROS) are down-regulated, which suggest that *Hv1* knockout shift the microglia/macrophage polarization from pro-inflammatory to anti-inflammatory status [184, 248, 250]. The decreased pro-inflammatory signals may be the reason for the reduction of periphery myeloid infiltration [248]. Taken together, Hv1 plays a key role in regulating microglia/macrophage activation, polarization, and infiltration after SCI.

Pathological acidosis in CNS is a common occurrence in traumatic brain injury (TBI) and SCI, which has a positive association with inflammation [183]. Intracellular and extracellular acidosis caused by TBI persisted for weeks and microglia proliferation and production of ROS were increased during the first week. When microglia were depleted by the colony-stimulating factor 1 receptor (CSF1R) inhibitor, PLX5622, TBI-induced extracellular acidosis, oxidative stress, and inflammation were markedly decreased during the acute stages of injury [183]. *Hv1*^-/-^ SCI mice model showed a significant reduction in tissue L-lactate after SCI compared to WT, which may be an indirect outcome of reduced microglia-derived signals to astrocytes [255]. It’s worth noting that although L-lactate contributes to tissue acidosis and its decrease in the *Hv1*^-/-^ SCI relates to a protective phenotype, it may also play beneficial roles during axon regeneration [184]. Taken together, Hv1 is a contributor to tissue acidosis after SCI.

SCI pathogenesis comprises neuronal loss, white matter injury, and reduced myelination, which are linked with increased Hv1 expression. A recent study using the *Hv1*^-/-^ mice model of SCI observed that Hv1 deficiency reduced neuronal apoptosis and NLRP3-inflammasome-mediated pyroptosis, improved axonal regeneration, and reduced motor deficits. SCI led to elevated ROS levels, whereas Hv1 deficiency down-regulated microglial ROS generation. In addition, ROS up-regulated neuronal pyroptosis and activated the NLRP3 inflammasome pathway, both of which were reversed by the addition of a ROS scavenger *in vitro* [184]. Besides, more pro-inflammatory mediators such as proIL-1β and caspase-11 have been demonstrated as priming signals for pyroptosis [184, 255]. Except for reduced neuronal loss, *Hv1* knockout also attenuated apoptosis of oligodendrocytes and ameliorated myelin loss [84, 184]. The mechanism of microglial Hv1 deficiency ameliorating demyelination may be the inhibition of ROS-mediated autophagy [84]. Taken together, Hv1 contributes to neuronal damage, oligodendrocytes apoptosis, and myelin loss after SCI.

Acute decrease in brain pH correlates with poor long-term outcomes in patients with TBI. Using a controlled cortical impact model in adult male mice, it is demonstrated that intracellular pH in microglia and extracellular pH surrounding the lesion site are significantly reduced and microglia proliferation and ROS production are also increased after injury. *Hv1*^-/-^ mice exhibited reduced pathological acidosis and inflammation after TBI, leading to long-term neuroprotection and functional recovery. The data, therefore, establish the microglial Hv1 proton channel as an important link that integrates inflammation and acidosis within the injury microenvironment during head injury [183].

In the SCI model of *Hv1*^-/-^ mice, Hv1 deficiency reduced neuronal apoptosis and NLRP3-inflammasome-mediated pyroptosis, improved axonal regeneration, and reduced motor deficits. The microglial ROS levels elevated after SCI were downregulated for the Hv1 deficiency. These results suggest that microglial Hv1 regulates neuronal apoptosis and NLRP3-induced neuronal pyroptosis after SCI by mediating ROS production [184].

Microglial Hv1 was significantly increased after spinal nerve transaction (SNT). *Hv1*^-/-^ mice display attenuated pain hypersensitivities and reduced microglial production of ROS, IFN-γ expression, and sequent astrocyte activation in the spinal cord after SNT compared with WT mice. These results demonstrate that the Hv1 proton channel contributes to microglial ROS production, astrocyte activation, IFN-γ upregulation, and subsequent pain hypersensitivities after SNT [47].

## 4. Clinical Translational Efforts of Targeting Microglial Hv1 in Myelin Repair

Microglia, brain-resident immune cells, respond rapidly to the pathological microenvironment and are upstream of the subsequent series of inflammatory waterfall responses, making it a star target for immunomodulatory therapy in demyelination-related diseases [14]. How to reverse the microglial inflammatory response from harmful to beneficial is an important topic in myelin repair. As the above discussion, the microglial Hv1 plays a complex role in demyelination and myelin repair. In traditional concepts, the inflammation response toward damage is harmful to tissue repair. However, important studies mentioned above also suggest the complex role of microglial inflammatory response and microglial Hv1 channel in myelin repair. Compared with NOX, Hv1 is a relatively simple homodimer, which may be a more tractable target in myelin repair [44]. Up to now, most studies about the microglial Hv1 have been conducted in *Hv1*^-/-^ mice, impossible for directed clinical applications [47, 53, 180-184, 194]. To realize the clinical application of targeting microglia Hv1 for myelin repair, a lot of effort has to be invested in finding Hv1-specific inhibitors and antibodies [52, 256-260]. There are a few compounds that are capable of inhibiting proton currents, including Zn^2+^ and other polyvalent cations, a tarantula toxin, guanidine derivatives, and clozapine and olanzapine [256-259, 261]. However, these Hv1 inhibitors have multiple targets, and show low potency of Hv1 inhibition [262]. Newly discovered novel HV1 inhibitors have not been applied to the CNS [260, 261]. Wang F et al. utilized stereotactic injection of rabbit anti-Hv1 antibody, and they found that neutralization of Hv1 promoted myelin debris clearance by microglia [52].

Although the existing attempts demonstrate the clinical translational perspective of targeting microglial Hv1 in myelin repair, there is a long way between bench and bedside. The primary issue is the search for specific and safe microglial Hv1 inhibitors or antibodies for myelin repair, which is the key to achieving precise immunomodulatory therapies. The Hv1 channel is expressed widely, both in microglia and peripheral immune cells, these two different sources of cells have different biological behaviors in the CNS [199]. The future direction may be exploring targeted or directional drug delivery systems that can permeate across the BBB to target microglial Hv1. More importantly, the treatment time windows should be clarified. Complex roles of microglial Hv1 channel closely related to disease-specific pathophysiological processes and microenvironments [14, 172]. Modulation of HV1 channels at different time points may cause opposite results [50, 64, 196].

### Conclusion and Perspectives

In summary, demyelination and remyelination are important factors in various neurological diseases. The voltage-gated proton channel Hv1 expressed by microglia plays a significant role in neuroinflammation, oxidative stress, and mitochondrial dysfunction associated with these diseases. Although there is still much to learn about the mechanisms of Hv1, current evidence suggests that it could be a promising therapeutic target for promoting myelin repair in various CNS disorders. Further studies are needed to deepen our understanding of Hv1 and its potential applications in the treatment of these conditions.
